# Maternal Sociodemographic Factors Are Associated with Methylphenidate Initiation in Children in the Netherlands: A Population-Based Study

**DOI:** 10.1007/s10578-020-01016-2

**Published:** 2020-06-21

**Authors:** K. Cheung, H. El Marroun, B. Dierckx, L. E. Visser, B. H. Stricker

**Affiliations:** 1 Department of Epidemiology, Erasmus Medical Center, PO Box 2040, 3000 CA Rotterdam, The Netherlands; 2 Health and Youth Care Inspectorate, Utrecht, The Netherlands; 3 Department of Child and Adolescent Psychiatry, Erasmus Medical Center, Rotterdam, The Netherlands; 4 Department of Pediatrics, University Medical Center Rotterdam, Erasmus Medical Center, Rotterdam, The Netherlands; 5 Department of Psychology, Education and Child Studies, Erasmus School of Social and Behavioural Sciences, Erasmus University Rotterdam, Rotterdam, The Netherlands; 6 Haga Teaching Hospital, The Hague, The Netherlands

**Keywords:** Child, Methylphenidate, Initiation, Medication, Attention deficit hyperactivity disorder

## Abstract

Multiple factors may contribute to the decision to initiate methylphenidate treatment in children such as maternal sociodemographic factors of which relatively little is known. The objective was to investigate the association between these factors and methylphenidate initiation. The study population included 4243 children from the Generation R Study in the Netherlands. Maternal sociodemographic characteristics were tested as determinants of methylphenidate initiation through a time-dependent Cox regression analysis. Subsequently, we stratified by mother-reported ADHD symptoms (present in 4.2% of the study population). When ADHD symptoms were absent, we found that girls (adjusted HR 0.25, 95%CI 0.16–0.39) and children born to a mother with a non-western ethnicity (compared to Dutch-Caucasian) (adjusted HR 0.42, 95%CI 015–0.68) were less likely to receive methylphenidate. They were more likely to receive methylphenidate when their mother completed a low (adjusted HR 2.29, 95%CI 1.10–4.77) or secondary (adjusted HR 1.71, 95%CI 1.16–2.54) education. In conclusion, boys and children born to a mother of Dutch-Caucasian ethnicity were more likely to receive methylphenidate, irrespective of the presence of ADHD symptoms.

**Electronic supplementary material:**

The online version of this article (10.1007/s10578-020-01016-2) contains supplementary material, which is available to authorized users.

## Introduction

Worldwide, 5% of children will develop or have symptoms of attention deficit hyperactivity disorder (ADHD) [1]. Stimulant medication is widely used for the treatment of ADHD of which methylphenidate is considered the first choice of pharmacological treatment [2]. However, not all children who are eligible for methylphenidate treatment receive the medication and conversely some children may receive methylphenidate when it is not needed [3]. When initiating treatment with methylphenidate, the risk of potential negative outcomes (such as major depression and suicidal behavior) may also be considered as shown in previous studies [4, 5]. Even though, there are clear guidelines with regard to ADHD treatment, symptom severity and functional impairment are not the sole determinants of treatment initiation. Numerous studies have explored other factors that might contribute to the use of stimulant medication in children diagnosed with ADHD [6, 7]. One of the factors known to be related to initiation with stimulant medication is the patient’s sex. Stimulants are more often prescribed to boys than girls, which can partly be explained by the fact that ADHD is more often diagnosed in boys than in girls. This may be reflected by the differences in ADHD symptoms between boys and girls [8–10]. Apart from child characteristics, the prescribing behavior of physicians as well as the availability of non-pharmacological treatment options to the family are important [11–13].

Not every child diagnosed with ADHD receives pharmacological treatment [14]. While symptom’s severity as well as non-response to non-pharmacological interventions play an important role in the initiation of pharmacological treatment [14], there may be other factors contributing to a family’s decision to visit a general practitioner or specialist (such as knowledge, apprehension regarding therapy and experience with the healthcare system), a child psychiatrist or to start medication [15, 16]. Children often rely on caregivers for support and management of chronic conditions involving taking medications [17]. Mothers are mostly considered as the primary caregiver, but the importance of maternal characteristics in determining methylphenidate treatment initiation should be studied further [18–20]. Previous studies showed that the utilization of ADHD medication may be influenced by sociodemographic factors such as ethnicity and socioeconomic status [9, 21]. These studies showed that medication was less frequently prescribed to ethnic minority groups. The relation between methylphenidate use and socioeconomic disadvantage could be associated with mental health problems in general [9]. However, these studies do not address the presence or absence of any ADHD related symptoms in these children, which may vary across the different sociodemographic groups [22, 23]. Furthermore, many studies about ADHD treatment are conducted in the US and they have a different healthcare system compared to Europe [24, 25]. A previous study in the Netherlands found that factors on the individual, family and GP practice level may determine ADHD medication prescription. They hypothesized that child characteristics would have the largest impact, followed by family and GP practice characteristics [26]. However, family characteristics that were included in this study was only limited to the number of children per household and previous ADHD medication use within their family. Other factors such as household income, education and ethnicity were not considered, but may play a role in the decision to start therapy with methylphenidate.

The objective of our study was to investigate the association between maternal sociodemographic and prenatal lifestyle factors in relation to child methylphenidate treatment initiation. Subsequently, we performed analyses stratified by the presence of (mother reported) clinically relevant ADHD symptoms in children using the determinants that were significantly associated with child methylphenidate prescription. We hypothesized that maternal sociodemographic factors are associated with the initiation of methylphenidate treatment in children. These maternal factors would account for variance in the treatment initiation, beyond the individual child characteristics. Apart from gender and the indication, family factors may have a large impact on the decision to start therapy with methylphenidate.

## Methods

### Design and Study Population

**Fig. 1:**
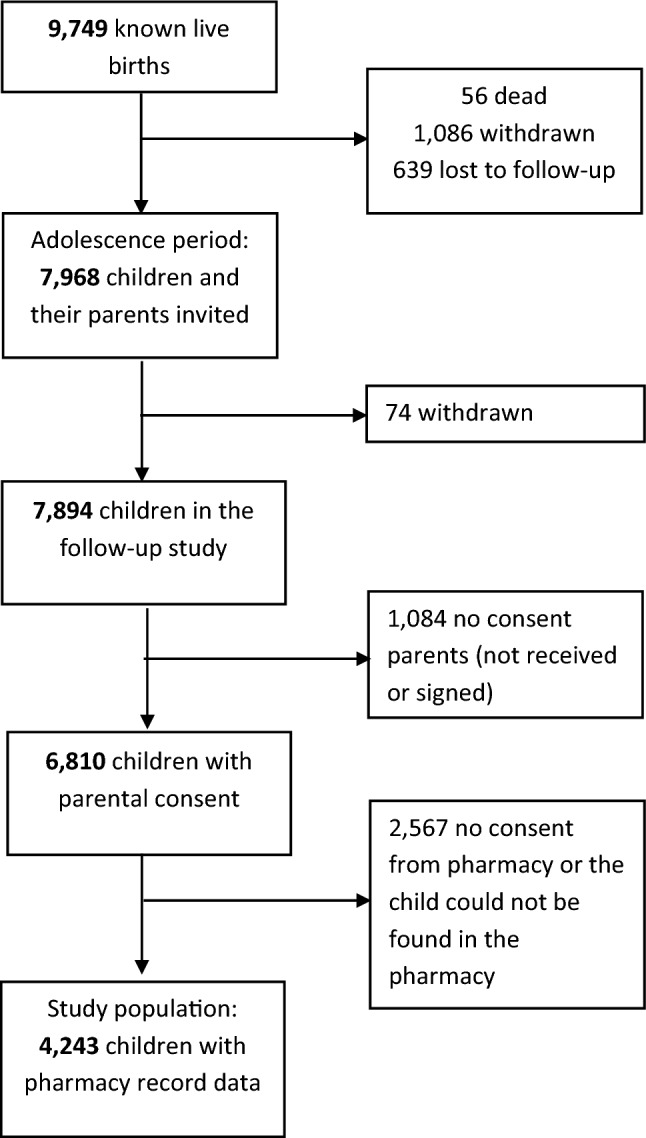
Selection study population

The study was conducted within The Generation R Study, which is a large prospective population-based cohort study investigating children’s health from fetal life onwards in Rotterdam, the Netherlands [27]. Pregnant women who were resident in Rotterdam and who had a delivery date between April 2002 and January 2006 were asked to participate in the study. In this cohort, detailed and extensive data collection has been conducted, which include questionnaires, interviews, and behavioral observations of children and their parents [27]. In addition, we retrieved pharmacy records from community pharmacies throughout Rotterdam, depending on where the child resided. In total, 9778 mothers were enrolled in the study and gave birth to 9749 live born children (Fig. 1). Of this group, 7896 children and their parents were invited to participate in the follow-up study (56 died; 1086 withdrawn from study; 639 lost to follow-up (no response to the invitation to participate in the follow-up study or moved to another city outside the defined study area). Children were excluded from the study if their parents chose to later withdraw from the study (n = 74). They were also excluded if no consent by their parents was provided (n = 1084) or because no consent was given by the pharmacist or the child could not be found in the pharmacy (n = 2567). Pharmacy records could be obtained from 4243 children. The Medical Ethics Committee of the Erasmus Medical Center approved all study procedures, and parents provided written informed consent.

### Child Methylphenidate Prescription

Pharmacy records of Generation R participants were collected based on the living area of their mothers (northern, southern, western of eastern part of Rotterdam). This information was used to determine at which pharmacies they may collect their medication. Pharmacists were contacted and asked for consent to retrieve the pharmacy records. Children may collect their medication at more than one pharmacy and these pharmacy records were all linked to one particular child. Pharmacy records of children may not be available because either their pharmacy or their parents did not give consent to retrieve these records or because the child’s pharmacy could not be found.

For 4243 individuals in The Generation R Study, all prescriptions which were filled at their pharmacy during the entire study period were gathered starting at birth. For each prescription we had the product name, anatomical therapeutic chemical (ATC) code [28], date of filling, number of delivered tablets/capsules, and prescribed daily number of doses. All study participants were followed from date of birth until a first prescription of methylphenidate or end of the study period at 1 September 2017, whichever came first. Information about the use of ADHD medication (N06BA) was obtained from the collected pharmacy dispensing records. Furthermore, information about the type of prescriber of the first methylphenidate prescription (e.g. general practitioner, specialist or at the hospital) was available in the electronic pharmacy records.

### Child ADHD Symptoms

The Child Behavior Checklist (CBCL/1.5–5 and CBCL/6–18) was used to obtain information about behavioral and emotional problems in children [29]. The CBCL is a questionnaire that was filled out by mothers when children were 1.5, 3, 5 and 9 years of age. The CBCL/6–18 was only used at the 9 years assessment. Children were classified as having ADHD symptoms in the borderline clinical range, when the cut-off score was above the 93^rd^ percentile [29, 30]. For the statistical analysis, we used the last questionnaire that was filled out by the mother prior to date of first methylphenidate prescription. The average time between the completion of the last CBCL and first prescription of methylphenidate was 2.9 years (SD: 3.6).

### Child and Maternal Characteristics

The following maternal lifestyle and demographic characteristics were considered as potential determinants for starting treatment with methylphenidate: maternal age at intake, ethnicity (Dutch-Caucasian or other Western, non-western, defined according to the classification of Statistics Netherlands [31]), household income (< €1200,  > €1200 and < 2000, > €2000 per month), education level (primary, secondary, higher), marital status (married, living together, no partner), alcohol use (yes/no), caffeine intake (yes/no) and smoking during pregnancy (yes/no) [32]. Maternal psychopathology in mid-pregnancy was assessed, using the Brief Symptom Inventory (BSI). This is a validated self-report questionnaire, which includes a spectrum of psychiatric symptoms. A weighted sum score above 0.75 means that clinically relevant psychopathology symptoms were present [33, 34]. In addition, we considered the use of selective serotonin reuptake inhibitors (SSRIs) during pregnancy (the most frequently used antidepressant in our study), which was obtained from pharmacy records and self-reported information [35]. This information from mothers was collected during pregnancy or at birth of their child. Finally, we also considered child’s sex as a potential determinant.

### Analysis

Child and family characteristics were presented for all children with pharmacy records, which also included the type of prescriber, age of first methylphenidate prescription and the life style factors of mothers. Second, we investigated which maternal lifestyle and demographic characteristics were related to methylphenidate initiation. For the main analyses, we calculated the hazard ratio (HR), with 95% confidence interval (CI) for each determinant associated with initiation of methylphenidate. We used a time-dependent Cox regression model, where non-methylphenidate users can serve as a control more than one time [36]. A time-dependent model was used, because the CBCL questionnaires were completed at different time points (1.5, 3, 5 and 9 years). For this analysis, we considered the presence of clinically relevant ADHD symptoms based on the CBCL questionnaire data that was completed prior to the date of first methylphenidate prescription. As determinants of methylphenidate treatment initiation, we considered all above-mentioned maternal and child characteristics in the univariate analysis. In addition, a multivariable Cox regression analysis was performed with the variables that were univariably associated. Separately, we tested the interaction between maternal education and ethnicity in the post-hoc analysis with an interaction term. Finally, we performed analyses stratified by clinically relevant ADHD symptoms (above 93rd percentile) using factors that were associated with child methylphenidate prescription in the main analysis.

First, we performed complete-case analyses. In the non-response analysis, we explored differences between mothers and children of responders and non-responders. In the sensitivity analysis, we performed the same analysis using multiple imputation of the covariates (where less than 30% was missing) using the expectation maximization algorithm to deal with missing data. Results were considered statistically significant at p < 0.05. Statistical analyses were performed using IBM SPSS Statistics for Windows, version 21.0 (IBM Corp., Armonk, NY).

## Results

### Child Methylphenidate Prescription

Pharmacy records were available for 4243 children. A total of 295 children were prescribed an ADHD medication, of whom 291 (99%) received methylphenidate. Due to the small number of children with other ADHD medication (dexamphetamine n = 3 and atomoxetine n = 1), we only analyzed data of children who started their treatment with methylphenidate. The average age of receiving the first methylphenidate prescription was 9.4 years. Of these children, 207 received their first methylphenidate prescription from a specialist (71%) and they were more often boys (n = 221, 75.9%).

### Demographic and Lifestyle Characteristics

In Table 1, the characteristics of the study population are shown. Half of the children who were included in our study were girls (n = 2066, 48.7%). Of all children whose mother filled in the CBCL questionnaires (n = 2070), 179 (8.6%) had ADHD symptoms above the cut off score. Furthermore, 1621 (38.2%) mothers had a non-western background, 2016 (47.5%) had a relatively high monthly household income (€ > 2000) and only a small percentage had a low education level (n = 361, 8.5%).

**Table 1:** Maternal and child characteristics of study population

Characteristic	N (%), n = 4243
Sex, girl	2066 (48.7)
ADHD symptoms present (CBCL by mother)^a^	
No	1891 (44.6)
Yes	179 (4.2)
Age of first methylphenidate prescription, mean (SD)*	9.4 (2.2)
Type prescriber *	
No methylphenidate prescription	3955 (93.2)
Specialist	209 (4.9)
General practitioner	37 (0.9)
Hospital	10 (0.2)
Maternal age (at intake) in years, mean (SD)	30.6 (5.2)
Parity	
1	2260 (53.3)
> 1	1983 (46.7)
Ethnicity mother	
Dutch-Caucasian and other western	2480 (58.4)
Non-western	1621 (38.2)
Household income	
< €1200	570 (13.4)
€1200–2000	571 (13.5)
€ > 2000	2016 (47.5)
Maternal education level	
No education/primary education	361 (8.5)
Secondary education	1708 (40.3)
Higher education	1747 (41.2)
Marital status	
Married	1921 (45.3)
Living together	1418 (33.4)
No partner	484 (11.4)
Smoking during pregnancy	
No	3056 (72.0)
Yes	596 (14.0)
Alcohol use during pregnancy	
No	1991 (46.9)
Yes	1322 (31.2)
Caffeine intake during pregnancy	
No	2105 (49.6)
Yes	1468 (34.6)
Maternal psychopathology (BSI)	
< 0.76	2770 (65.3)
0.76 and higher	485 (11.4)
SSRI use during pregnancy	
No	1798 (42.4)
Yes	36 (0.8)

Numbers are given in numbers (percentages) unless stated otherwise. The numbers of the missing values are not shown in this table, but are as follows: type prescriber 22 (0.5%); reported ADHD symptoms 2173 (51.2%); ethnicity 142 (3.3%); household income 1086 (25.6%); maternal education 427 (10.1%); marital status 420 (9.9%); smoking during pregnancy 591 (14%); alcohol use during pregnancy 930 (21.9%); caffeine intake during pregnancy 670 (15.8%); maternal psychopathology 988 (23.3%) and SSRI use during pregnancy 2409 (56.8%)

^*^The information about the type of prescriber was only provided of children who received methylphenidate (n = 291)

^a^ Clinically relevant ADHD symptoms: Children were classified as having ADHD symptoms in the borderline clinical range when their cut-off score was above the 93^rd^ percentile. *BSI* Brief Symptom Inventory, *N* number of children, *SD* standard deviation, *SSRI* selective serotonin reuptake inhibitors. All numbers are given in percentages or mean (SD)

### Determinants of Methylphenidate Treatment Initiation

In the multivariable analysis (Table 2), we found that girls were less likely to receive methylphenidate compared to boys (adjusted HR 0.34, 95%CI 0.24–0.49). As expected, children were more likely to receive a methylphenidate prescription when mothers reported clinically relevant child ADHD symptoms (adjusted HR 8.67, 95%CI 6.25–12.02). We observed that children were more likely to start treatment with methylphenidate when mothers completed secondary school (adjusted HR 1.52, 95%CI 1.09–2.12) compared to mothers with a higher education level. Furthermore, a non-western ethnicity of mothers was associated with a lower likelihood of methylphenidate treatment (adjusted HR 0.41, 95%CI 0.28–0.60) compared to a Dutch-Caucasian or other western background. Finally, the interaction between maternal education and maternal ethnicity was found to be significant (P: 0.001). Children whose mother received a secondary education as compared to a higher education in the western group were more likely to receive methylphenidate (HR 1.93, 95%CI 1.45–2.58). In the non-western group, no significant association was found (no/ primary education HR 0.70, 95%CI 0.36–1.38; secondary education HR 0.71, 95%CI 0.42–1.20).

**Table 2:** Maternal and child factors associated with methylphenidate treatment initiation

Characteristic	Cases, N (%) (n = 291)	Crude HR, 95%CI	Adjusted HR, 95%CI (cases, n = 180)
Sex child			
Boy	221 (75.9)	Ref	Ref
Girl	70 (24.1)	***0.31 (0.24–0.41)***	***0.34 (0.24–0.49)***
ADHD symptoms present (CBCL by mother)^a^			
No	128 (44.0)	Ref	Ref
Yes	78 (26.8)	***9.06 (6.71–12.23)***	***8.67 (6.25–12.02)***
Maternal age at baseline, years			
< 25	44 (15.1)	Ref	
25–30	85 (29.2)	0.87 (0.59–1.27)	
31–36	123 (42.3)	0.77 (0.54–1.10)	
> 36	39 (13.4)	0.81 (0.52–1.27)	
Parity			
1	154 (52.9)	Ref	
> 1	137 (47.1)	1.02 (0.80–1.29)	
Ethnicity mother			
Dutch-Caucasian and other western	201 (69.1)	Ref	Ref
Non-western	82 (28.2)	***0.60 (0.46–0.79)***	***0.41 (0.28–0.60)***
Household income			
< €1200	29 (10.0)	0.67 (0.44–1.00)	
€1200–2000	38 (13.1)	0.89 (0.61–1.28)	
€ > 2000	150 (51.5)	Ref	
Maternal education level			
No education/primary	20 (6.9)	0.91 (0.56–1.49)	1.04 (0.52–2.06)
Secondary	138 (47.4)	***1.37 (1.06–1.78)***	***1.52 (1.09–2.12)***
Higher	106 (36.4)	Ref	ref
Marital status			
Married	113 (38.8)	Ref	
Living together	106 (36.4)	1.29 (0.98–1.70)	
No partner	40 (13.7)	1.44 (0.99–2.10)	
Alcohol use during pregnancy			
No	124 (42.6)	Ref	
Yes	100 (34.4)	1.23 (0.94–1.62)	
Smoking during pregnancy			
No	196 (67.4)	Ref	Ref
Yes	55 (18.9)	***1.48 (1.09–2.03)***	1.02 (0.70–1.48)
Caffeine intake during pregnancy			
No	143 (49.1)	Ref	
Yes	97 (33.3)	1.04 (0.80–1.35)	
Maternal psychopathology (BSI)			
< 0.76	178 (61.2)	Ref	
0.76 and higher	33 (11.3)	1.06 (0.72–1.56)	
SSRI use during pregnancy			
No	132 (45.4)	Ref	
Yes	4 (1.4)	1.58 (0.55–4.53)	

A time-dependent model was used where the ADHD scores of the last completed CBCL questionnaire prior to methylphenidate prescription was considered. In the adjusted model we included all univariably associated determinants. The number of non-methylphenidate users are not presented in the table due to the time component of the model where non-methylphenidate users can serve as a control more than once

^a^Clinically relevant ADHD symptoms: Children were classified as having ADHD symptoms in the borderline clinical range when their cut-off score was above the 93^rd^ percentile ADHD indicates attention deficit hyperactivity disorders, *BSI* Brief Symptom Inventory, *CBCL* Child Behavior Checklist, *CI* confidence interval, *HR* hazard ratio, *N* number of children, *SD* standard deviation, *SSRI* selective serotonin reuptake inhibitor

### Stratification by ADHD Symptoms

#### Absence of ADHD Symptoms

Table 3 shows that girls without clinically relevant ADHD symptoms were less likely to receive a methylphenidate prescription compared to boys (adjusted HR 0.25, 95%CI 0.16–0.39). Children with mothers of a non-western background were less likely to receive a methylphenidate prescription compared to those with a Dutch-Caucasian background (adjusted HR 0.42, 95%CI 0.26–0.68). Furthermore, we found that children were more likely to receive a methylphenidate prescription when mothers completed a lower education compared to those who completed high education (no education/ primary education adjusted HR 2.29, 95%CI 1.10–4.77 and secondary education adjusted HR 1.71, 95%CI 1.16–2.54).

**Table 3:** Maternal and child factors associated with methylphenidate treatment initiation stratified by the presence and absence of clinically relevant ADHD symptoms

Characteristic	Cases, N	Crude HR, 95% CI	Adjusted HR, 95% CI^b^	Crude HR, 95% CI	Adjusted HR, 95% CI^b^
		ADHD symptoms absent		ADHD symptoms present^a^	
Sex					
Boy	159	Ref	Ref	Ref	Ref
Girl	47	***0.26 (0.17–0.39)***	***0.25 (0.16–0.39)***	***0.54 (0.32–0.89)***	***0.53 (0.32–0.90)***
Ethnicity					
Dutch-Caucasian and other western	149	Ref	Ref	Ref	Ref
Non-western	55	***0.51 (0.33–0.77)***	***0.42 (0.26–0.68)***	***0.41 (0.25–0.68)***	***0.45 (0.26–0.77)***
Maternal education					
No education/primary	16	1.20 (0.61–2.35)	***2.29 (1.10–4.77)***	0.38 (0.14–1.03)	0.60 (0.21–1.70)
Secondary	101	1.33 (0.90–1.95)	***1.71 (1.16–2.54)***	0.92 (0.55–1.54)	1.10 (0.65–1.86)
Higher	74	Ref	Ref	*Ref*	*Ref*

A time-dependent model was used where the ADHD scores of the last completed CBCL questionnaire prior to methylphenidate prescription was considered. In the adjusted model we included all determinants. The number of non-methylphenidate users are not presented in the table due to the time component of the model where non-methylphenidate users can serve as a control more than one time

^a^Clinically relevant ADHD symptoms: Children were classified as having ADHD symptoms in the borderline clinical range when their cut-off score was above the 93rd percentile

^b^Corrected for time between completion of CBCL and first methylphenidate prescription. ADHD indicates attention-deficit hyperactivity disorder; *CBCL* child behavior checklist, *CI* confidence interval, *HR* hazard ratio, *N* number of children

### Presence of ADHD Symptoms

When child ADHD symptoms above the cut-off were reported, we found that girls were less likely to receive a methylphenidate prescription than boys (adjusted HR 0.53, 95%CI 0.32–0.90) (Table 3). Children of non-western mothers were less likely to receive a methylphenidate prescription compared to children of mothers with a Dutch-Caucasian or other western background (adjusted HR 0.45, 95%CI 0.26–0.77).

### Sensitivity Analyses

In the multivariable analysis with imputed data we found similar results: direction and size of the effect estimates overall did not change much. We found that girls were less likely to receive methylphenidate (adjusted HR 0.31, 95%CI 0.21–0.47) or when they had a mother with a non-western background (adjusted HR 0.48, 95%CI 0.31–0.75). Children were more likely to receive a methylphenidate prescription when they had ADHD symptoms above the cut-off reported by their mothers (adjusted HR 10.12, 95%CI 6.95–14.74). However, the association between methylphenidate initiation and a low maternal education was non-significant (no/primary education adjusted HR 2.01, 95%CI 0.94–4.28; secondary education adjusted HR 1.37, 95%CI 0.94–2.01).

### Non-response Analyses

For the variables that were included in the analyses, less than 30% were missing, except for SSRI use during pregnancy (56.8%) and the reported clinically relevant ADHD symptoms (51.2%). The non-response analysis showed no significant differences between children with and without information for the maternal characteristics. However, we found that 2173 women did not complete the CBCL questionnaires at all or did not complete the questionnaire prior to the first prescription of methylphenidate. A dropout analysis on this variable showed that the missing of the CBCL questionnaire was not significantly associated with sex, but it was associated with ethnicity (P < 0.001), maternal education (P < 0.001) and methylphenidate initiation (P < 0.001).

The analysis in the group without information on ADHD symptoms (missing information) showed similar results for sex (girls adjusted HR 0.49, 95%CI 0.30–0.83) and ethnicity (non-western background adjusted HR 0.61, 95%CI 0.36–1.03). For maternal education, we found that a lower education was significantly associated with a decreased risk of methylphenidate use compared to higher education (no education/ primary HR 0.31, 95%CI 0.11–0.91).

Pharmacy records were not available for half of the cohort participants (results not shown in table). For children of whom no pharmacy records were available, we found that their mothers were significantly younger (maternal age 29.6 vs 30.6, P < 0.001), had a lower household income (20.6% < 1200 vs 18.1%, P < 0.02), were lower educated (12.0% no education/primary education vs 9.5%, P < 0.001), more often did not have a partner (15.6% no partner vs 12.7%, P0.001), used less alcohol during pregnancy (35.4% vs 39.9%, P < 0.001), smoked more during pregnancy (19.7% vs 16.3%, P < 0.001) and had a lower caffeine intake during pregnancy (61.9% vs 58.9%).

## Discussion

### Main Findings

In the current study, we found that several child and maternal sociodemographic factors were related to methylphenidate treatment initiation. In our study, we were able to examine these determinants stratified by the presence and absence of clinically relevant ADHD symptoms. Our findings show that methylphenidate was more frequently prescribed to boys than girls. It also shows that children of mothers with a non-western background were less likely to receive a methylphenidate treatment than children of mothers with a Dutch-Caucasian or other western background. These findings are both in line with results that have been shown in previous studies [9, 10, 37]. However, the previous studies only addressed the association with sociodemographic factors in patients with an ADHD diagnosis. In our study, we found that even when no ADHD symptoms were reported by mothers, boys and children born to mothers with a western ethnic background were still more likely to receive methylphenidate. Furthermore, we found that a low and secondary maternal education (compared to a high education) was associated with methylphenidate prescription in children without reported symptoms. A previous study of Russel et al., found no significant association between maternal education and medication use in children in a UK population [8]. This could also be explained by the differences in the educational system of the Netherlands and the UK. It is also possible that they did not find the association as they only explored the sociodemographic factors of medication use among children with ADHD.

### Explanations for These Findings

First, sex differences with regard to use of methylphenidate or other stimulants is probably related to the diagnosis of ADHD, which is more common in boys than girls [38]. This could be explained by the fact that ADHD was once thought to be a predominantly male disorder [39]. Boys with clinically ADHD, present more outwardly signs of ADHD, such as hyperactive and impulsive behavior, while girls present more inwardly signs, such as inattentiveness and low self-esteem (attention deficit disorder, ADD)[40–42]. This may both lead to boys being diagnosed with ADHD more often as well as earlier initiation of pharmacological treatment as shown previously [8]. However, in our study we observed that girls are still less likely to receive methylphenidate irrespective of the presence of ADHD symptoms. It is possible that girls may be less qualified as their symptoms are not considered severe enough [43]. Parents may find it more difficult to cope with the hyperactive and impulsive behavior, which is more prevalent in boys and are therefore more likely to seek help by visiting the GP. This could also be the reason why boys are more likely to be diagnosed and treated with medication. It could imply that girls with ADHD symptoms are undertreated while on the other hand boys without ADHD may be overtreated [44]. It has also been noted that treatment programs are mainly focused on behavior management, which has a higher priority among boys than girls [45]. The difference in the symptom profile among boys and girls may be a reason why boys are more frequently prescribed methylphenidate than girls [46].

Second, we showed that children without ADHD symptoms of mothers who only had limited education (no education, primary and secondary education) were more likely to receive a methylphenidate prescription. On the other hand, no significant association with maternal education was observed in children with ADHD symptoms. This finding might be interpreted that as long as the child has ADHD, there is no problem with inequity. When it comes to children without a diagnosis, there is inequity in treatment. However, these results should be carefully interpreted due to the association of missing information of ADHD symptoms and maternal education. Nevertheless, a previously published study showed that a low maternal education was associated with less involvement in the decision-making of medication initiation in children. Parents may not have sufficient knowledge about ADHD and feel that it is necessary to initiate methylphenidate when this is not thoroughly discussed with them [47]. Furthermore, we found that children of mothers with a western ethnic background who received a lower education, were more likely to receive methylphenidate than children whose mother received a high education. It may seem that only mothers with a western ethnic background with a low education are less likely to be involved. This association was not found in the non-western group. This may suggest that mothers with a non-western background, irrespective of educational level, are treated differently than mothers with a western ethnic background.

Third, children with ADHD problems and parents from ethnic minority groups may have less access to healthcare or less communication with healthcare professionals due to a language barrier [23, 24, 48–51]. They may also receive less appropriate diagnoses or treatments as the symptoms observed for these disorders may differ across ethnic groups, and may differ from what clinicians are trained to expect [52, 53]. ADHD problems are also often recognized by teachers when children are more hyperactive than others. However, not all parents may consider hyperactivity as a behavioral problem as some parents may have a positive attitude towards a child with high energy [54]. This view and recognition of ADHD related problems may vary across different ethnic groups. Furthermore, ethnic minority families may also be less likely to recommend medication and may prefer behavioral therapy over stimulant medication as found in previous studies [55, 56]. The findings of the current study may reflect cultural differences, knowledge and perceptions about ADHD and its pharmacological treatment. These cultural differences may include behavioral expectations and tolerance, attachment, attention, personality and many other aspects of parenting. It does not only influences the environment in which a child with ADHD functions, but also in the way this child is understood and treated by its parents [57]. It may also influence the decision to visit a GP or not as some parents may not recognize emotional and behavioral problems as such. A previous study showed that significantly higher scores of hyperactivity were given in China and Indonesia compared to other countries such as the US [57]. However, it is also possible that parents prefer to seek for other ways to cope with the hyperactivity or emotional problems of their child as they do not want to put their child on chronic medication.

These characteristics may be associated with certain medication types [58, 59, 60]. However, we have tested this with other medications such as SSRIs, antihistamines and NSAIDs. We found that children born to a mother with a non-western background or a low education were more likely to receive these medications (see supplemental Table 1). As prescribing of methylphenidate was less likely in girls and the non-western group, as can be seen in Table 3, this suggests that symptoms of these children may less readily considered as a problem.

### Strengths and Limitations

Strengths of our study are the relatively large population-based cohort, its prospective design, independent registration of dispensed medicines, and the multi-ethnic nature of the sample which limit the chance of selection and information bias. Treatment initiation was based on pharmacy dispensing records, which is more accurate in terms of dispensation date than information on prescription medication from medical records or questionnaires as medication can be prescribed but not collected at pharmacies. However, our study also has some limitations. One of the limitations is that we had to rely on questionnaires filled out by mothers to assess the presence of ADHD symptoms. These mother reports are considered valuable as they provide more insight into the perspective of the mothers with regard to their child’s behavior as ADHD symptoms are not always recognized as such across different demographic groups. However, not all mothers completed the CBCL questionnaire, which is also considered as an important limitation as we were not able to assess the presence of ADHD symptoms in these groups. Information bias may have occurred if the association between maternal characteristics or sociodemographic factors and treatment initiation is different for responders and non-responders, but this is difficult to ascertain. Nevertheless, the stratified analysis in the group without information on ADHD symptoms showed similar results for sex and ethnicity, except for maternal education. Further research is needed to assess the role of maternal education in the treatment initiation of methylphenidate, in particular in relation to the presence of ADHD symptoms. Another limitation is that pharmacy records were not available for half of the participants of The Generation R study as not all pharmacists provided consent to obtain the dispensing records from their pharmacies. As shown in the results, we found that mothers of children without pharmacy records differed on several aspects. Despite this selection bias, we observed similar results in our study compared to the available literature showing an association between initiation of methylphenidate and ethnicity, maternal education level and smoking during pregnancy [47, 61]. Furthermore, no information about other treatments (e.g. behavioral therapy) was available. Therefore, we were not able to assess if specific demographic groups were receiving behavioral therapy or no therapy at all. Finally, information about maternal characteristics were not available for each child, but the non-response analysis showed no significant differences between both groups.

## Summary

We assessed the association between child and maternal factors and the initiation of methylphenidate treatment. Additionally, we also assessed the association in the stratified analysis with mother-reported ADHD symptoms. The results of the study showed that apart from the child’s sex, the mother also plays an important role in the treatment initiation. We also found that even when no ADHD symptoms were reported, boys and children born to a mother of Dutch-Caucasian ethnicity or a low maternal education were still more likely to receive methylphenidate treatment. The difference in symptom profile among boys and girls may affect the decision to start pharmacological treatment. Furthermore, the decision of parents to start treatment with methylphenidate may depend on their knowledge and perceptions about ADHD and pharmacological treatment, which may vary among different ethnic groups. Considering these findings, it is important for healthcare professionals to be aware of these differences and take these into account when deciding on initiating treatment with methylphenidate. As parents play an important role, they should always be involved in the decision-making process of treatment initiation with methylphenidate. Reasons for treatment initiation in children without ADHD symptoms should be further investigated.

## Electronic supplementary material

Below is the link to the electronic supplementary material.

Electronic supplementary material 1 (DOCX 43 kb)
