# Supplementary material for: Maternal Sociodemographic Factors Are Associated with Methylphenidate Initiation in Children in the Netherlands: A Population-Based Study
Source: Child Psychiatry Hum Dev. 2020 Jun 21;52(2):332–42. doi: 10.1007/s10578-020-01016-2 (PMC7973638; doi:10.1007/s10578-020-01016-2)
Supplement: Supplementary file 1 — Electronic supplementary material 1 (DOCX 43 kb) [file 10578_2020_1016_MOESM1_ESM.docx]

**Supplemental material**

**Supplemental figure 1.** **Maternal and child factors associated with methylphenidate treatment initiation in patients without reported ADHD symptoms**

*Abbreviation: ADHD indicated attention deficit hyperactivity disorder*

**Supplemental figure 2.** **Maternal and child factors associated with methylphenidate treatment initiation in patients with reported ADHD symptoms**

*Abbreviation: ADHD indicated attention deficit hyperactivity disorder*

**Supplemental table 1. Characteristics associated with use of SSRIs, antihistamines or NSAIDs.**

| **Characteristic** | **SSRI, OR, 95%CI** | **Antihistamines, OR, 95%CI** | **NSAIDs, OR, 95%CI** |
| --- | --- | --- | --- |
| Gender |  |  |  |
| Boy | ref | ref | ref |
| Girl | 1.44 (1.33-1.56) | 0.95 (0.95-0.96) | 0.95 (0.94-0.97) |
| Ethnicity |  |  |  |
| Western | ref | ref | ref |
| Non-western | 1.26 (1.15-1.38) | 1.15 (1.14-1.16) | 1.15 (1.13-1.17) |
| Education |  |  |  |
| No education/  primary | 2.16 (1.93-2.40) | 1.51 (1.49-1.53) | 1.72 (1.67-1.77) |
| Secondary | 0.41 (0.37-0.45) | 1.27 (1.25-1.28) | 1.34 (1.31-1.36) |
| Higher | ref | ref | ref |

*Abbreviations: CI indicates confidence interval; NSAIDs, nonsteroidal anti-inflammatory drugs; OR, odds ratio; SSRI, selective serotonin reuptake inhibitors.*
